# Fast and ultrafast thermal contrast amplification of gold nanoparticle-based immunoassays

**DOI:** 10.1038/s41598-022-14841-3

**Published:** 2022-07-26

**Authors:** Yilin Liu, Li Zhan, Joseph Kangas, Yiru Wang, John Bischof

**Affiliations:** 1 Department of Mechanical Engineering, University of Minnesota, Minneapolis, MN 55455 USA; 2 Department of Biomedical Engineering, University of Minnesota, Minneapolis, MN 55455 USA

**Keywords:** Surface plasmon resonance, Biomedical engineering, Techniques and instrumentation

## Abstract

For highly sensitive point-of-care (POC) diagnostics, we explored the limit of thermal contrast amplification (TCA) reading of gold nanoparticles (GNPs/mm^2^) at test regions in immunoassays. More specifically, we built and compared fast (minute scale) and ultrafast (seconds scale) TCA setups using continuous-wave (CW) and ms pulsed lasers, respectively. TCA improved the limit of detection (LoD) for silica-core gold nanoshells (GNSs) preloaded in nitrocellulose (NC) membrane as model lateral flow immunoassays (LFAs) by 10- to 20-fold over visual reading. While the ultrafast TCA led to higher thermal signals, this came with a twofold loss in LoD vs. fast TCA primarily due to noise within the infrared sensor and a necessity to limit power to avoid burning. To allow higher laser power, and therefore amplification fold, we also explored transparent glass coverslip substrate as a model microfluidic immunoassay (MIA). We found the ultrafast TCA reading of GNS-coated coverslips achieved a maximal signal amplification (57-fold) over visual reading of model LFAs. Therefore, ultrafast TCA-MIA is promising for ultrasensitive and ultrafast diagnostics. Further advantages of using TCA in MIA vs. LFA could include lower sample volume, multiplexed tests, higher throughput, and fast reading. In summary, TCA technology is able to enhance the sensitivity and speed of reading GNPs (GNPs/mm^2^) within both LFAs and MIAs.

## Introduction

Point-of-care (POC) disease diagnosis, especially paper-based lateral flow immunoassay (LFA) tests, has been widely used to screen for disease infections such as SARS-CoV-2 and influenza. The prevalence of these tests is mainly due to their rapidity to result (10–15 min), low cost, and simplicity of use. However, their sensitivity can vary largely across different studies, ranging from as low as ~ 30% to over 90%^1–4^.

To boost the sensitivity of LFAs, efforts have been focused on sample enrichment and LFA improvement, including signal amplification and assay optimization^5–8^. Among the various signal amplification methods, thermal contrast amplification (TCA) has been used in our previous studies, which relied on an infrared (IR) detector to record the photothermal signals of the laser-excited gold nanoparticle (GNP) labels captured at the test line of LFAs^7,9^. In cohort validation studies, TCA was shown to significantly reduce false negatives of commercial LFAs by identifying sub-visual positives for group A *Streptococcus* and influenza A and B^10,11^. As characterized by serial antigen-dilution studies, TCA was able to improve the detection sensitivity by about 8- to 16-fold over visual reading, depending on the laser power used^9,12,13^. The applications of TCA were summarized in a table from a previous publication^10^. Note that the amplification fold eventually saturated with increasing laser power due to the amplified noise along with signals^5,12^. As such, the sensitivity improvement was limited by nonspecific binding (NSB) of GNPs in the assay^5,12^. Fortunately, this limitation can be addressed by assay optimization coupled with signal amplification to reduce NSBs, thus achieving fM—aM detection sensitivity to SARS-CoV-2 spike protein in buffer and human nasopharyngeal wash^5,12^. However, the maximal signal enhancement by TCA for an optimal LFA with minimal NSBs is unknown.

However, adding signal amplification steps can also increase the diagnosing time, which can be a drawback to POC use. To cut down the time of reading, various improvements in signal amplification methods have been investigated^5–8^. In the case of TCA, a fast reader was developed with a continuous-reading algorithm and a continuous-wave (CW) laser; it enabled < 1 min reading per test while maintaining a sensitivity improvement similar to that of a previous discrete but-slow reading (~ 15 min) format^14^. To achieve the speed limit, it is also interesting to explore the potential of ultrafast TCA reading using a ms pulsed laser which would enable a complete readout within seconds.

Based on the above questions, we established an ultrafast 1064 nm ms pulsed laser TCA reader to compare to the existing fast CW laser (1064 nm) TCA reading approach and to see if further signal enhancement and reading speed can be achieved. For maximal thermal amplification, different substrates for the immunoassay were studied, including the nitrocellulose (NC) membrane used in traditional LFA and glass coverslip as a model for microfluidic immunoassay (MIA). In each case, the substrates (NC membrane and glass coverslip) were pre-coated with silica-core gold nanoshells (GNSs) which have a plasmon peak near 1064 nm. Results of proof-of-concept experiments showed that the maximal amplification fold (57-fold) was achieved by ultrafast TCA reading of GNP-precoated coverslip at the maximal energy output of the pulsed laser over visual reading of GNSs pre-loaded NC membrane (i.e., model LFAs). Even higher amplification fold would be achieved if further improvements to IR sensor and noise reduction were made on the ultrafast TCA. It is also envisioned that the ultrafast TCA would improve the sensitivity of rapid diagnostic testing during an epidemic or pandemic in clinics and distributed testing sites.

## Results and discussion

### Setting up TCA readers with CW vs. pulsed lasers

To achieve ultra-high signal amplification fold on the GNP labels, the TCA system can be improved by increasing the laser energy fluence. During laser irradiation, the heat generation of a GNP, $\dot{{Q}_{GNS}}$, can be estimated as

1 $$\dot{{Q}_{GNP}}={C}_{abs}\bullet {I}_{0},$$

where ${C}_{abs}$ is GNP’s absorption cross section (unit: ${\mathrm{mm}}^{2}$), and ${I}_{0}$ is the energy fluence of laser irradiation (unit: $\mathrm{W}\cdot{\mathrm{mm}}^{-2}$). Increasing ${I}_{0}$ creates a higher photothermal response from GNPs ($\dot{{Q}_{GNS}})$, which could help lower TCA’s detection limit of GNPs in LFA. In most previous studies, a CW laser at 532 nm was used in TCA and the regular irradiation power on LFAs was set as ~ 25 mW^10,11,13,15^. The measured diameter of the laser spot on LFA was about 0.1 mm^13^, whose average input energy fluence, ${I}_{0}$, was estimated as 3.2 $\mathrm{W}\cdot{\mathrm{mm}}^{-2}$ (Table 1).

**Table 1:** Comparison of heat generation per gold nanoparticle ($\dot{{Q}_{GNP}}$) when irradiated by continuous-wave (CW) or pulsed laser. Laser wavelength: $\lambda$; absorption cross section: ${C}_{abs}$.

Laser irradiation				Gold nanoparticles			$\dot{{Q}_{GNP}}$ (W)	$\dot{{Q}_{GNP}}$ increase (fold)	References
Types	$\lambda$ (nm)	Intensity, ${I}_{0}$ ($\mathrm{W}\bullet {\mathrm{mm}}^{-2}$)	Power/energy	Geometry	Diameter (nm)	${C}_{abs}$ (${\mathrm{nm}}^{2}$)			
CW	532	3.2	25 mW	Sphere	30	1.45E3	4.6E−9	1	^9,13,15^
					60	1.11E4	3.6E−8	8	^15^
					100	2.08E4	6.7E−8	14	^12,15^
					120	2.35E4	7.5E−8	16	^14^
CW	1064	12.7	100 mW	Silica-cored gold shell	Core: 198 Shell: 242	1.01E4	1.3E−7	28	This work
Pulsed (170 V)	1064	22.3	1.41 J				2.3E−7	49	
Pulsed (400 V)	1064	955.4 (maximum)	60.64 J				9.6E−6	2080	

The ${C}_{abs}$ of a GNP at the wavelength of the corresponding laser was calculated by Mie theory^18^. All laser irradiation conditions listed in table were within glass coverslip’s tolerance (i.e., without burning or damaging by laser irradiation). The thermal tolerance of nitrocellulose membrane was much lower than glass coverslip, whose maximal pulsed laser setting was about 170 V. Examples of undamaged GNS spots in nitrocellulose membrane after such pulsed laser irradiation was shown in Supplementary Fig. S8b. More discussion on substrates’ difference in laser power tolerance is provided in Supplementary Sect. S4.

To maximize the photothermal response of GNPs, the traditional CW laser was upgraded to a pulsed laser with higher energy fluence. Here, a 1064 nm Nd:YAG laser (iWeld 980 Series, 120 J, LaserStar Technologies, FL, USA) was used to provide a high-energy singular millisecond pulse, as shown in Supplementary Fig. S1a. As calibrated, the highest laser pulse energy was 60.64 J within 20 ms^16^. For a 2 mm spot, the energy fluence from the pulsed laser was up to 955.4 $\mathrm{W}\cdot{\mathrm{mm}}^{-2}$, about 300-fold higher than that in previous studies^10,11,13,15^. To maximize $\dot{{Q}_{GNP}}$ under the same laser irradiation, the GNS was chosen over other GNPs, such as gold nanorods (about 90 nm in length and 15 nm in width) which also absorb strongly at 1064 nm, because GNS has larger ${C}_{abs}$ than other GNPs as characterized in a previous study^17^. Table 1 compares the $\dot{{Q}_{GNP}}$ of different GNP-laser settings. The GNS-pulsed laser (400 V) setting has the highest heat generation which can be as high as 2080-fold of that for the 30 nm gold nanosphere (GNSp)-CW laser (25 mW) setting. Thus, it was chosen to test the limit of TCA. However, less than maximum pulsed laser intensity (22.3 $\mathrm{W}\cdot{\mathrm{mm}}^{-2}$) was used to test GNS-loaded NC membrane (model LFA) since it was prone to burn under more intensive irradiation.

**Figure 1:**
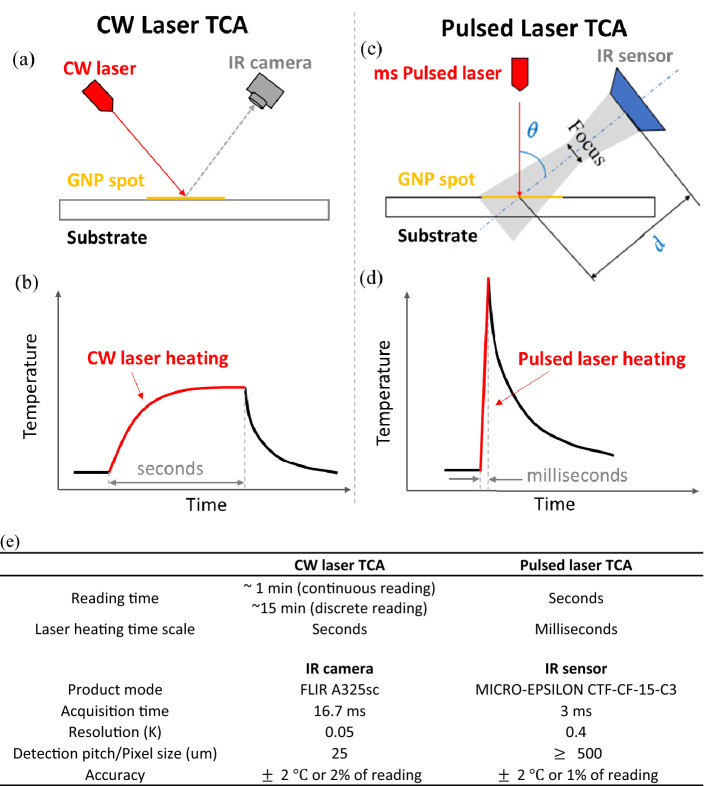
TCA readers equipped with continuous-wave (CW) laser vs. pulsed laser. (**a**) Arrangement of the laser path, IR camera, and testing platform, such as a substrate coated with gold nanoparticle (GNP) spot, in the CW laser TCA reader. (**b**) Schematic record of temperature response of a GNP spot heated by CW laser. (**c**) Arrangement of the laser path, IR sensor, and testing platform in the ultrafast TCA reader equipped with an ms pulsed laser. The gray area was the field of view of the IR sensor, which depends on the alignment parameters, *d* and $\theta$ (detailed in Supplementary Sect. S1). (**d**) Schematic record of temperature response of a GNP spot read by pulsed laser. (**e**) Comparison of reading time, laser heating time scale, and temperature measurement products in ultrafast TCA vs. CW laser TCA with continuous and discrete reading algorithms.

To test the limit of TCA, both TCA readers equipped with CW laser and pulsed laser were set up to compare their limits of detection (LoDs) for GNPs precoated in NC membrane and on coverslips as immunoassay models. Their schematic setup is shown in Fig. 1a,c. More details on CW laser TCA can be referred to our previous work^9,13^. Details of ultrafast TCA setup and characterization are provided in Supplementary Sect. S1. As compared between Fig. 1b,d, different lasers enable different heating intensity and speed. When heating a GNP spot with an ms pulsed laser, the heating energy from pulsed laser was confined within the laser spot which, in turn, enabled a much higher temperature increase than CW laser heating (detailed in Supplementary Sect. S4). The temperature increase of a GNP spot can be done within ms by pulsed laser heating while CW laser would need many seconds to heat the spot. As summarized in Fig. 1e, faster reading can be achieved with the pulsed laser ultrafast TCA (seconds) than CW laser TCA either with discrete or continuous reading algorithms (1–15 min) as detailed in previous work^9,13,14^. Additionally, different temperature measurement products (IR camera vs. sensor) were used to fit with the lasers as summarized in Fig. 1e.

In addition to lasers, GNP-loaded substrates being irradiated also impact the thermal responses. In general, substrates with lower thermal mass and higher tolerance for laser intensity against thermal damages will achieve higher thermal signals. Table 2 lists three substrates (NC membrane, plastics, and coverslip) that can be potentially used for immunoassays and testing the limit of TCA. NC membrane (widely used in LFAs) and coverslip were chosen as substrates to be tested in this study since they had significant differences in both thermal mass normalized by volume and maximum temperature without thermal damage.

**Table 2:** Comparison of thermal mass and maximum temperature without thermal damage between substrates for immunoassays with thermal contrast amplification.

Substrates	Normalized thermal mass ($\mathrm{J}\bullet {\mathrm{m}}^{-3}{\mathrm{K}}^{-1}$)	Maximum temperature ($^\circ \mathrm{C}$)
Nitrocellulose	4.0E+05	~ 180 (pyrolysis)^19^
Plastics (acrylic)	1.8E+06	~ 290 (pyrolysis)^20^
Coverslip (glass)	1.5E+06	~ 1400 (melting)

### Testing the limit of TCA with CW or pulsed lasers using model LFAs

**Figure 2:**
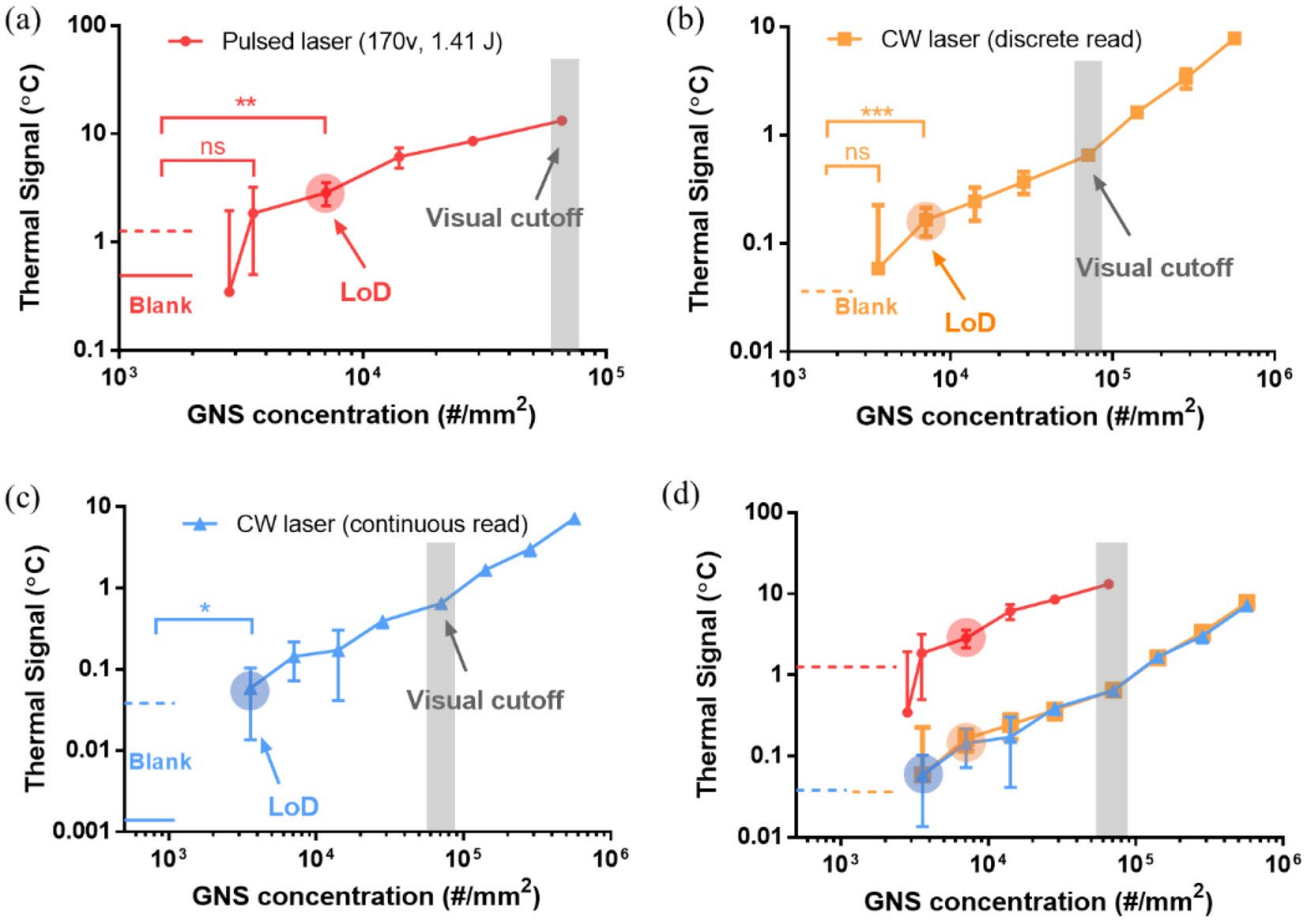
Reading gold nanoparticles in nitrocellulose (NC) membrane as model lateral flow immunoassays (LFAs) by TCAs with continuous wave (CW) laser *vs.* pulsed laser. NC membrane was precoated with diluted silica-cored gold nanoshells (GNSs) as model test regions in lateral flow immunoassays. (**a**) Thermal signals from ultrafast TCA reading with a pulsed laser (22.3 $\mathrm{W}\cdot{\mathrm{mm}}^{-2}$, 170 V, 1.41 J, 3 ms) (red). (**b**) Thermal signals from CW laser TCA reading with a discrete reading algorithm (yellow). (**c**) Thermal signals from fast TCA reading with CW laser and continuous reading algorithm (blue). (**d**) Comparison of these thermal signals from different TCA readings. Round shadows: limits of detection (LoDs) for GNSs. Square shadow (gray): visual cutoff to read GNS spot in NC membrane (model LFA). Statistical significance is indicated with asterisks: ns: p > 0.05; *p < 0.05; **p < 0.01. The GNS concentration in NC membrane was the projected surface concentration = volumetric concentration $\times$ membrane thickness.

To test the limit of TCA, we compared thermal signals of the (pulsed laser) ultrafast TCA with CW laser TCA when reading the same model LFAs (GNS-loaded NC membrane) as seen in Fig. 2. The UV–vis-NIR extinction spectrum of the GNS is shown in Supplementary Fig. S3. The intensity output of the pulsed laser was set at 22.3 $\mathrm{W}\cdot{\mathrm{mm}}^{-2}$ (Table 1) to avoid thermal damage to NC membrane, whose thermal signals are shown in Fig. 2a. For CW laser TCA, both traditional discrete reading and continuous reading (i.e., fast reading) were applied and results were plotted in Fig. 2b,c, respectively. The CW laser intensity was set at 12.7 $\mathrm{W}\cdot{\mathrm{mm}}^{-2}$ (100 mW, Table 1), nearly twofold lower than that from ultrafast TCA. Compared with visual reading of model LFAs, TCA readings showed a 10- to 20-fold reduction in LoD for GNSs loaded in NC membrane, as shown in Fig. 2a–c. The ultrafast TCA had higher thermal signals than CW laser TCA for the controlled GNS concentrations, as compared in Fig. 2d. However, it also had much higher background noise for the blank NC membrane (i.e., without GNSs). We speculate that this is due to the limitation of the IR sensor. Ideally, the acquisition time of the IR sensor should be at least tenfold smaller than the pulse width (3 ms) to ensure the accuracy and consistency of the signal acquisition. Unfortunately, in our case, the IR sensor, which was chosen based on its small size to fit into laser chamber and price consideration, had a comparable acquisition time of 3 ms (Fig. 1e) despite the claim that it could show interpolated temperature at 1 ms interval; this may contribute to some noise or inconsistency in the reading. In contrast, the CW laser TCA had a much faster temperature acquisition (16.7 ms) than the laser heating time scale (seconds), thus with high reading consistency. Perhaps, as a result, the current ultrafast TCA setup did not show an apparent benefit in signal amplification to read model LFA compared to the fast TCA. The lowest LoD was achieved by the fast TCA reading (i.e., using CW laser and continuous reading algorithm), and was twofold lower than those from ultrafast TCA and the other discrete reading algorithm. Future optimization may consider a more advanced IR sensor, although a higher cost is expected. Alternatively, increasing laser’s pulse width can reduce the impact of IR sensor’s inadequate sampling, which can also enhance thermal signals with a significant increase in laser energy fluence. Since NC membrane was prone to pyrolysis and burn under intensive laser heating (Table 2), another assay substrate (i.e., glass) was considered to test the limit of TCA in the next section.

### Changing substrates of immunoassay for higher thermal contrast amplification fold

**Figure 3:**
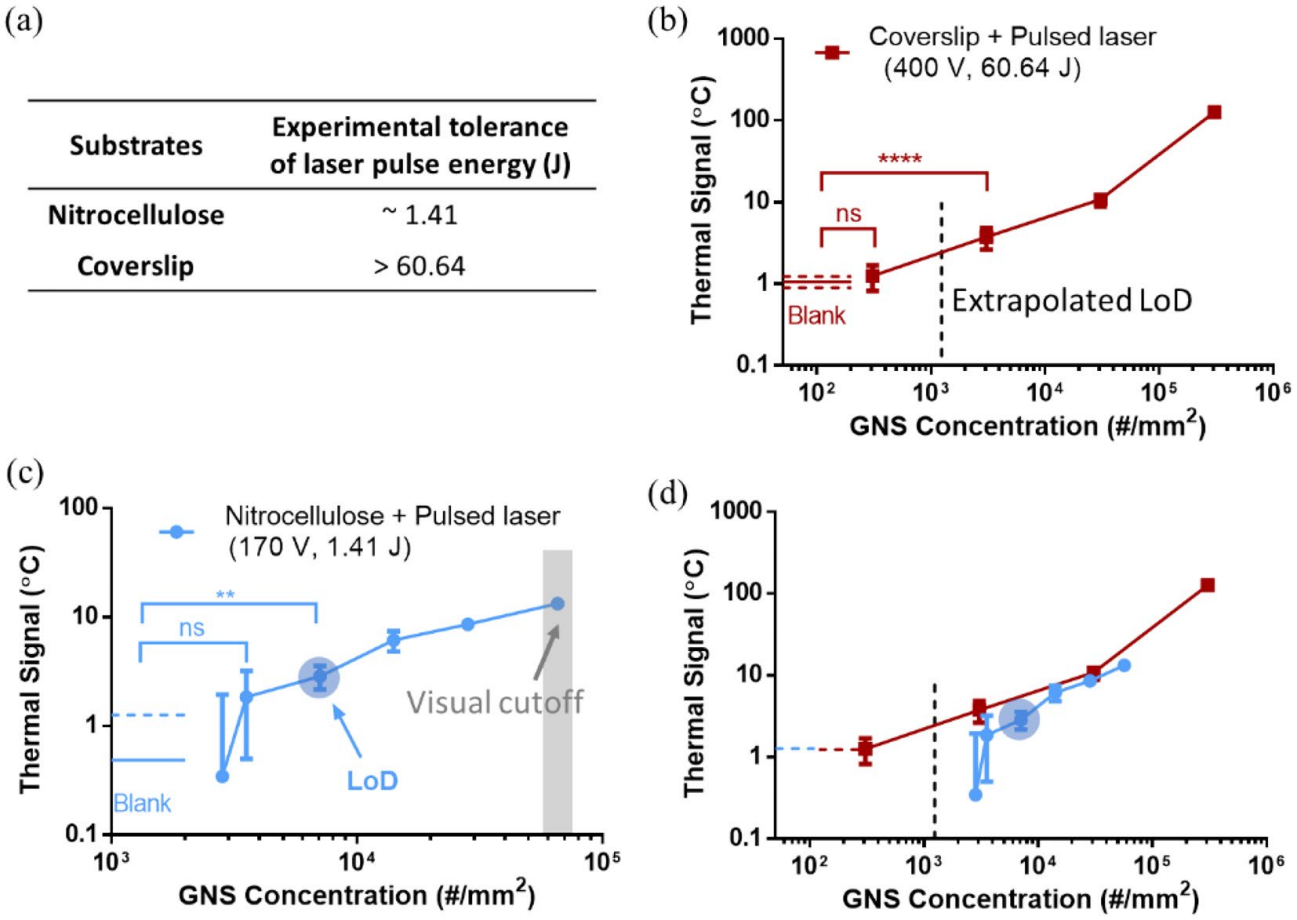
Testing the limit of thermal contrast amplification (TCA) by improving substrates for ultrafast TCA reading. Thermal signals were measured through ultrafast TCA reading silica-cored gold nanoshells (GNSs) precoated in nitrocellulose (NC) membrane and on coverslips at the same projected surface concentrations as model test regions in immunoassays. (**a**) Experimental tolerance of laser pulse energy by the tested GNS-NC membrane and GNS-coverslip systems. (**b**) Thermal signals from GNS-coverslips with maximal laser pulse energy (60.64 J) over 20 ms. (**c**) Thermal signals from GNS-NC membrane with laser pulse energy at 1.41 J over 3 ms to avoid thermal damage. (**d**) Comparison of these thermal signals from different substrates. Blue round shadow: limits of detection (LoDs) for GNSs in NC membrane. Square shadow (gray): visual cutoff to read GNS spot in NC membrane. Dashed line: extrapolated LoD for GNSs on coverslip by IUPAC metric. All the coverslip cases were subvisual. Statistical significance is indicated with asterisks: ns: p > 0.05; *p < 0.05; **p < 0.01; ***p < 0.001; ****p < 0.0001. The GNS concentration in NC membrane was the projected surface concentration = volumetric concentration $\times$ membrane thickness.

For even higher signal amplification, proof-of-concept measurement was conducted by TCA reading of GNSs pre-coated on a glass coverslip as a model MIA, which can tolerate much higher irradiation intensity than either paper or plastic (see Table 2 and Fig. 3a). To maximize the thermal signals in measurement, the maximal energy output of the pulsed laser (400 V, 60.64 J, 20 ms pulse width, and 2 mm spot size, ${I}_{0}=$ 955.4 $\mathrm{W}\cdot{\mathrm{mm}}^{-2}$) in ultrafast TCA was applied to detect GNSs on the coverslips in Fig. 3b. A stricter metric (IUPAC, see “Methods”) was applied to extrapolate the LoD for GNSs on coverslip by ultrafast TCA reading rather than ANOVA analysis which was used as default for other measurements. To understand the benefit of coverslip, its thermal signals were compared with those of model LFAs with NC membrane read by ultrafast TCA but at lower pulse energy (1.41 J) to avoid thermal damage (Fig. 3c). Unlike model LFAs, the GNS-coverslips in Fig. 3b were all subvisual due to poor visual contrast, while the visual cutoff of model LFAs was shown in Fig. 3c. Regarding ultrafast TCA reading as compared in Fig. 3d, the coverslips had higher thermal responses than model LFAs for the same GNS concentrations. The thermal LoD for GNSs on coverslip was also lower (~ 57-fold) than the visual LoD for model LFAs. This suggests that increasing laser pulse energy enabled higher thermal responses, which compensated for the large thermal mass of coverslip. Since coverslip has better thermal tolerance, 20 ms pulse was applied, which was ~ 6.7-fold longer than the acquisition time of IR sensor (3 ms). Thus, the sensor sampling issue that may have influenced readings in the model LFA case (Fig. 3c) was likely not an issue here (Fig. 3b). Further modeling and discussion on substrate comparison for TCA are provided in Supplementary Sect. S4 to potentially achieve even higher thermal signals and thus better signal amplification from TCA reading. Certainly, finding a sensor that can operate under even shorter pulses with improved signal-to-noise will also help.

**Figure 4:**
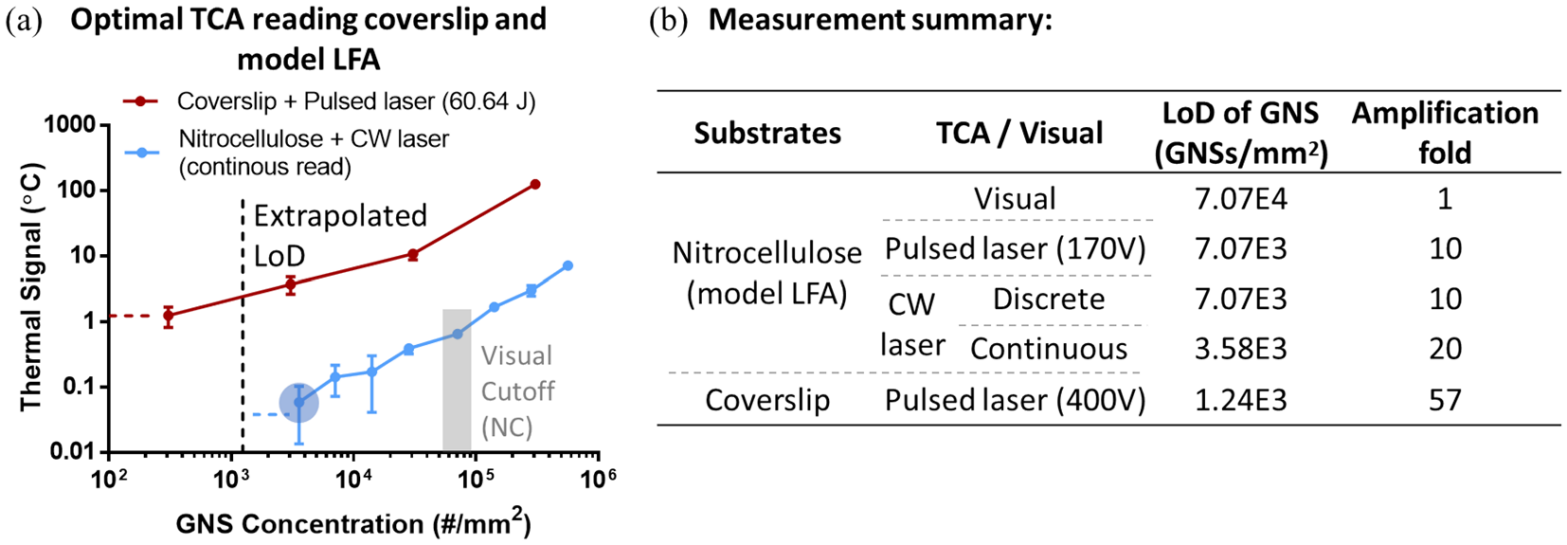
(**a**) Comparison of thermal signals from diluted silica-cored gold nanoshells (GNSs) precoated in nitrocellulose (NC) membrane (model LAF) and on coverslips as model test regions in immunoassays when being read by their respective optimal thermal contrast amplification (TCA) systems. Model LFA was read by fast TCA (i.e., continuous-wave (CW) laser with a continuous reading algorithm) while coverslips were read by ultrafast TCA at maximal energy output. Blue round shadow: limits of detection (LoDs) for GNSs in NC membrane. Square shadow (gray): visual cutoff to read GNS spots in NC membrane. All the coverslip cases were subvisual. Dashed line: extrapolated LoD for GNSs on coverslip by IUPAC metric. (**b**) Summary of the LoDs for GNSs precoated in/on different substrates (i.e., NC membrane or coverslip) and read by different TCA systems. Their corresponding amplification folds were calculated by comparing them with visual cutoff for reading GNSs in NC membrane. For NC membrane, GNS concentration was the projected surface concentration = volumetric concentration $\times$ membrane thickness.

Figure 4a compared thermal signals from GNS-coverslip and GNS-NC membrane (or model LFA) when being read by their respective optimal TCAs. The LoD for GNSs in the coverslip case (1.24E3 GNSs/mm^2^) was still about 2.85-fold lower than that of the NC membrane case. This further proved that increasing the laser fluence can improve thermal response and signal amplification fold via TCA reading, and thus the sensitivity of immunoassays. Figure 4a also showed that the background noise of blank samples for ultrafast reading of GNS-coverslip was around 1 °C, much higher than GNS-NC membrane with fast TCA reading, which may set the major limit to an even lower LoD. This noise might be due to the system error of the ultrafast TCA, absorption of laser energy by glass, etc. For even greater MIA sensitivity enhancement by TCA, future efforts would be needed to reduce the background noise.

To summarize, Fig. 4b shows the LoDs for GNSs measured on various substrates (NC membrane vs. coverslip) when being read by different TCA systems (CW laser vs. pulsed laser). The signal amplification folds were normalized by the visual cutoff of reading model LFAs, which is a conventional readout format for commercial LFAs. The coverslip and ultrafast TCA with maximal pulsed laser energy output had the maximal signal amplification (57-fold), followed by the model LFA with fast TCA reading (20-fold). When reading model LFAs, the discrete reading by CW laser TCA showed a similar amplification fold (tenfold) to the ultrafast TCA. It is also expected that the amplification fold by ultrafast TCA could be further improved by reducing the background noise and/or using a better IR sensor (faster response), despite the higher cost and other changes in TCA setup. For future ultrafast TCA-MIA applications, the consideration of assay kinetics and design was also discussed in Supplementary Sect. S5 apart from signal amplification. Overall, TCA is able to enhance signals for both LFAs and MIAs. MIA with TCA is promising for future ultrasensitive POC diagnostics, although further improvement in reducing background noise will be needed if further signal amplification is needed or required.

## Conclusion

To increase both signal amplification and speed of reading with TCA, we established an ultrafast TCA reader by upgrading the traditional CW laser to a pulsed laser and the IR camera to an IR sensor. Proof-of-concept experiments were conducted to evaluate how sensitively the ultrafast TCA reader could detect GNSs in NC membrane as model LFAs and on coverslips as model MIAs. These results were compared with CW laser TCA reading of both substrates. We found that the limit of TCA reading in model LFA format had 10- to 20-fold improvement in LoD for GNSs than visual reading. The fast TCA had the best signal amplification fold, with a detection sensitivity twofold better than the ultrafast TCA likely due to a risk of burning with higher laser energy, and limitations in the IR sensor sampling at fast pulses (3 ms acquisition time vs 3 ms applied pulsed laser). On the other hand, we found that the limit of TCA reading in the MIA format at longer pulse widths (20 ms) and higher laser pulse energy to be 57-fold lower than visual reading of model LFAs. Therefore, TCA is capable to enable ultra-high signal amplification to different immunoassay formats and MIA with ultrafast TCA is promising for future ultrafast and ultra-sensitive POC diagnostics.

## Methods

### Preparing diluted GNS spots on substrates

The concentrated stock GNSs (nanoComposix, Inc., product number: GSGH980) with known concentration were diluted in either 65% glycerol solution or DI water to prepare test dots in NC membrane and on coverslips, respectively. Each GNS spot (i.e., test dot) in NC membrane was made by pipetting a drop of 0.6 $\upmu$L GNS solutions (in 65% glycerol) onto the NC membrane, followed by air-drying overnight. The 65% glycerol solution helped minimize the coffee ring effect during the spot drying^15^. The example of GNS spots on NC membrane was shown in Supplementary Fig. S8a. The spot size was measured to calculate the projected surface concentration of the GNS dots in NC membrane.

The schematic procedures to prepare GNS spots on coverslips are shown in Supplementary Fig. S9. A large polydimethylsiloxane (PDMS, SYLGARD^®^ 184) stamp (~ 5 mm thick) was made by mixing the base and curing agent at 10:1 weight ratio, pouring the mixture into a petri dish, de-bubbling in a vacuum, and heating at 70 $^\circ \mathrm{C}$ for 6 h. The PDMS stamp was then cut into small pieces (~ 1 cm square), and a 5 mm hole was punched onto each piece. The PDMS piece was cleaned by sonication in DI water for 3 min and dried in N_2_ flow. Meanwhile, a coverslip A (size: 2 cm × 3 cm, 0.1 mm thickness) was cleaned by sonication in DI water for 3 min, dried in N_2_ flow, and pretreated by oxygen plasma cleanser at 150 mtorr of oxygen gas and 40 W of power for 1 min. Immediately after pretreatment, the coverslip A was attached with a pre-cleaned PDMS piece to make the PDMS well. To prepare the GNS spot, 20 $\mathrm{\mu L}$ diluted GNS solution (in DI water) with known concentration was pipetted into the PDMS well. The well was sealed by Parafilm immediately and seated horizontally in a refrigerator to let the GNSs settle. After sitting overnight, the well was unsealed and dried at room temperature. To align the GNS spot with irradiation spot for TCA reading, another coverslip B was used to mark the well’s position (i.e., GNS spot’s position) on the coverslip A since the GNS spots can hardly be identified visually after being prepared (Supplementary Fig. S9). The PDMS stamp was gently removed before testing the GNS spots by TCA readers.

### CW laser TCA reading

The GNS spots from NC membrane and coverslips were read across by a CW TCA reader, using either continuous or discrete reading algorithms. A 1064 nm CW laser (Spectrum Stabilized Laser Module, Model #: I1064SR0300B, Innovative Photonic Solutions Inc.) was used, and the power reaching the substrates was 100 mW. Details of the two reading algorithms and other components were described elsewhere^13,14^. Briefly, in discrete reading, the scanning was made point-by-point at 0.125 mm distance. For each point, the pre-waiting time was 1 s before laser heating for 3 s, followed by cooling for 3 s without laser irradiation. In continuous reading, the laser heating spot was moving at a constant speed (0.1 mm/s) across the GNS spots. The area under the temperature curves when reading across a GNS spot was calculated as thermal signals. The LoD for GNSs by CW laser TCA reading was determined by Analysis of Variance (ANOVA) analysis of thermal signals obtained from blank and low-GNS samples with a p-value < 0.05.

### Ultrafast TCA setup and reading

The setup and characterization of the ultrafast TCA reader are provided in Supplementary Sect. S1. During ultrafast TCA reading, a single laser pulse was fired onto the GNP spot, and the temperature response was recorded. To demonstrate that the IR sensor (MICRO-EPSILON CTF-CF-15-C3) was providing millisecond resolution, we recorded the temperature change when a laser pulse (pulse width = 5 ms) hit the GNPs on a glass slide. Supplementary Figure S2f shows that temperature took ~ 5 ms to reach the maximum, which generally fit the laser pulse width, and then dropped after the laser pulse. The temperature difference between the peak temperature and the steady-state temperature before pulsed laser heating was calculated as the temperature increase. To acquire the thermal signal of a GNS spot, both the GNS spot and its nearby blank regions were read by the ultrafast TCA, as shown in Supplementary Fig. S10. The difference in temperature increase between the GNP spot and its nearby blank regions was calculated as the thermal signal.

The LoD for GNSs in NC membrane by ultrafast TCA reading was determined by the same metric as used for CW laser TCA (i.e., ANOVA analysis). The LoD for GNSs on coverslip was determined by the IUPAC metric due to the significant difference (p-value < 0.0001) in thermal signals between the low concentration sample and the blank sample (Fig. 3b). Detailed calculation procedures have been reported in previous work^13,21^. Briefly, the thermal signal threshold by the IUPAC metric was set as 3 times the standard deviation above the average thermal signal from blank samples. The LoD for GNSs was then determined as the corresponding GNS concentration to the thermal signal threshold, based on a linear fitting of the obtained thermal signals versus different GNS concentrations.

## Supplementary Information

Supplementary Information.

## Data Availability

The data are available from the corresponding author on request.
