# Supplementary material for: Fast and ultrafast thermal contrast amplification of gold nanoparticle-based immunoassays
Source: Sci Rep. 2022 Jul 26;12:12729. doi: 10.1038/s41598-022-14841-3 (PMC9321340; doi:10.1038/s41598-022-14841-3)
Supplement: Supplementary file 1 — Supplementary Information. [file 41598_2022_14841_MOESM1_ESM.docx]

**Supporting Information**

**Fast and Ultrafast Thermal Contrast Amplification of Gold Nanoparticle-Based Immunoassays**

Yilin Liu^1^, Li Zhan^1,a^, Joseph Kangas^1,a^, Yiru Wang^1^, John C. Bischof*^,1, 2^

^1^ Department of Mechanical Engineering, University of Minnesota, Minneapolis, MN 55455, USA

^2^ Department of Biomedical Engineering, University of Minnesota, Minneapolis, MN 55455, USA

^a^ These authors contributed equally to this work.

Email of the corresponding author: bischof@umn.edu

# S1. Ultrafast Thermal Contrast Amplification (TCA) Reader

Figure 1a shows the pulsed laser for an ultrafast TCA reader. The pulsed laser has a chamber in which the laser was fired from the top (Figure S1a), therefore the temperature reader needs to fit into the laser chamber. After a thorough search of commercial IR sensors, the MICRO-EPSILON IR sensor (model CTF-CF-15-C3, Figure S1b) was selected due to its small size, wide temperature range (-50 ~ 975 °C), good temperature resolution (±2 °C), ultrafast response time (3 ms), and, importantly, small focus spot diameter (~0.5 mm at minimum). To achieve maximal temperature readout, the alignment of the IR sensor, laser, and testing region on the platform (*e.g.*, a gold nanoparticle spot on a substrate) in Figure S1c were characterized and optimized below prior to use.

In ultrafast TCA reading, the temperature readout of the IR sensor was the average temperature of the detection area on the testing platform. This detection area depended on the distance (*d*) and the angle ($\theta$) between the sensor and the surface being tested (Figure S1c). To characterize it, we proposed a “hot wire” method, as shown in Figure S2. Specifically, for a given *d* and * * the sensor detection area was an ellipse with length *L* and width *w*. A “hot wire,” a narrow paper strip (0.5 mm width) painted black, was fixed on a plastic sample holder, as shown in Figure S2b. A CW laser (WorldStar, TECGL-30G-520-A) with a beam diameter of ~1 mm was used to irradiate the “hot wire” to generate a higher temperature than that of environment, making it detectable by the IR sensor. The sensor was mounted on XY linear stages which can move across the “hot wire.” The temperature and position were recorded. When moving the detection area (*i.e.*, moving the sensor) to cover the high-temperature “hot wire,” we observed an increase in temperature. After the moving detection area across the “hot wire,” the temperature dropped to the minimum (Figure S2c). The distance that the sensor traveled with a noticeable temperature increase over environmental background was the length or width of the detection area. The detection area of the IR sensor was characterized using the “hot wire” method, as presented in Figure S2d,e. At * = *° and *d* = 8.5 mm, the detection area was focused with a minimum diameter equal to ~0.5 mm, which matched well with the claimed value from the manufacturer. As expected, with increasing *θ*, the length (*L*) of the detection area increased, while the width (*w*) stayed the same under a fixed distance (*d*).

When applying ultrafast TCA reading, the alignment between laser path, IR sensor, and the testing area (gold nanoparticle, GNP, spot on a substrate) on the sample holder (Figure S1b) was adjusted as above for maximal temperature reading. Since the laser came from the top, the IR sensor was tilted at a certain angle (*θ* ~ 45 $^{\circ}$) to avoid blocking the light path. The alignment between the IR sensor and the GNP spot from a testing sample was first optimized by adjusting $d$ and $\theta$ for a maximal temperature readout when the GNP spot was heated by a CW laser (WorldStar, TECGL-30G-520-A) from the top. The sensing stage was then put into the laser chamber and the GNP spot was aligned with the laser spot by the laser’s built-in alignment assistance. Thus, the alignment between these 3 components was optimized.

**
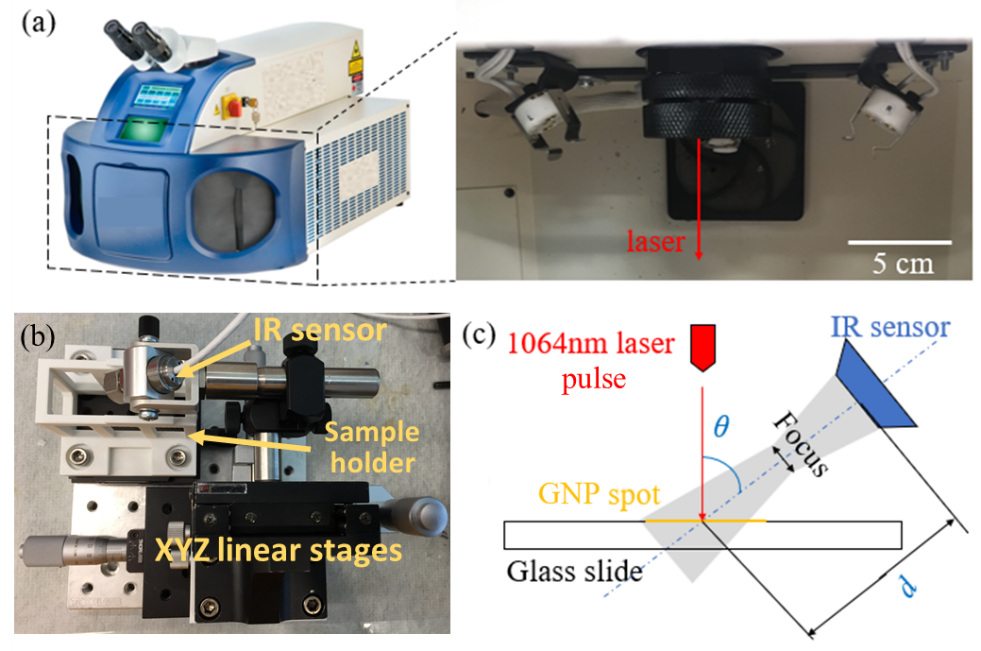
**

**Figure S1**. **Setting up an ultrafast TCA reader.** (a) Pulse laser used for ultrafast TCA reader and the close-up view of the laser chamber. (b) Top view of the sensing part, which was put into the laser chamber in (a) to assemble the ultrafast TCA reader. (c) Arrangement of the laser path, IR sensor, and testing platform, such as a gold nanoparticle (GNP) spot on a substrate, in the ultrafast TCA reader. The gray area was the field of view of the IR sensor, which depends on the alignment parameters, *d* and $\theta$.


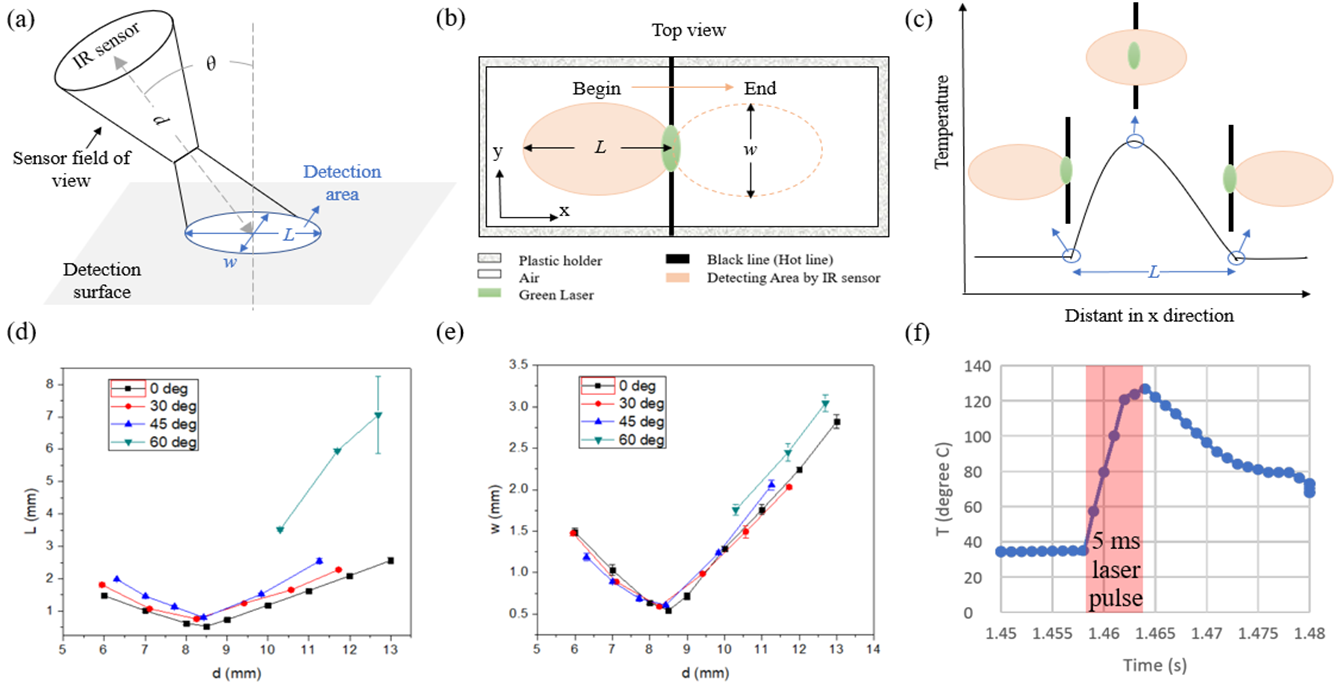


**Figure S2.** **Characterizing ultrafast TCA.** (a) The detection area of the IR sensor was a function of the d and $\theta$. The detection area was featured by *L* and *w*. (b) “Hot wire” setup to measure the detection area of the IR sensor. (c) As the sensor detection area moved across the “hot wire,” the recorded temperature increased and then decreased. The distance that the IR sensor traveled with an obvious temperature increase over the background was the *L* or *w* of the detection area. (d) Measured length *L* of detection area versus *d* at different $\theta$. (e) Measured width *w* of the detection area versus *d* at different $\theta$. (f) Example of the IR sensor recording a millisecond temperature change when a GNP spot on a coverslip was irradiated by a 5 ms laser pulse.

# S2. More Information on Gold Nanoparticles and TCA reading

**Figure S3**. The UV-vis-NIR extinction spectrum of silica-cored gold nanoshells provided by nanoComposix, Inc.

# S3. Fast TCA Reading GNSs in Nitrocellulose *vs.* on Coverslip

To understand the optical impact of substrates on GNSs’ photothermal responses, GNS-precoated nitrocellulose (NC) membrane and coverslip were first tested and compared by applying the same fast TCA reading, where the GNS-NC membrane set-up served as model lateral flow immunoassay (LFA). Figure S5a shows that the GNSs on the coverslip showed much lower (over 10-fold difference) thermal signals than those of model LFA with NC membrane for the same projected surface concentration (= volumetric concentration$\times$membrane thickness). To understand the cause of this large difference, we conducted modeling to show how the substrates influence GNSs’ thermal responses in section S4.


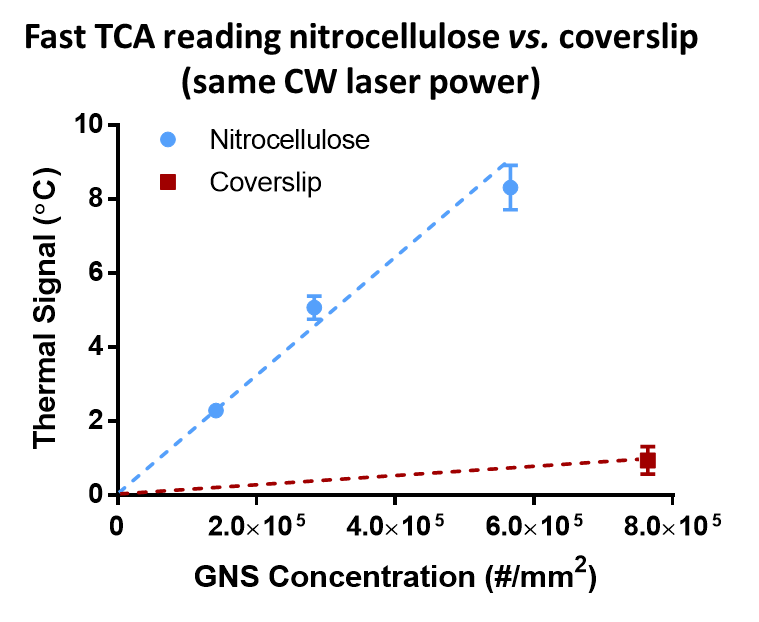


**Figure S4**. Measured thermal signals from silica-cored gold nanoshells (GNSs) precoated in NC membrane and on coverslips with the same projected surface concentration when read by the same fast thermal contrast amplification (TCA) reading (*i.e.*, continuous-wave laser and a continuous reading algorithm).

# S4. Simulation on TCA Reading GNSs in Nitrocellulose vs. on Coverslip

## S4.1 Comparing Predicted TCA Reading of GNSs in NC membrane *vs.* Coverslip

To understand the effects of substrates on TCA signals, simulation was conducted to compare thermal responses from GNP-loaded NC membrane (model LFA) and coverslips under the same CW laser irradiation. Specifically, we modeled the photothermal responses of 30 nm gold nanospheres (GNSps) during CW laser (25 mW, $1/e^{2}$radius of 0.05 mm) heating when loaded in NC membrane or on a coverslip at the same projected surface concentration, expecting to apply the comparison to other GNPs. Figure S5a shows that the GNSps had much higher total absorption rates (~30-fold) of laser energy when loaded in NC membrane than on a coverslip. The high scattering property of the porous membrane (see Table S1) substantially increased light-GNSp interaction chances by scattering and changing the light path. In contrast, GNSps on a coverslip had only a single chance to interact with the incident light regardless of reflection and thus showed less total absorption rate. Furthermore, to provide detectable IR signals, the GNSps need to heat the substrates. Even with the same heat source input, the coverslip would still have a lower temperature increase due to its larger thermal mass (normalized by area) than membrane (~7.5-fold difference) as shown in Figure S5a. As a result, the GNSp-NC membrane system had higher temperature increase than the GNSp-coverslip during the same CW laser heating, as shown in Figure S5b. Figure S5c also predicted the steady-state temperature increase under different laser power for the modeled systems. As expected, when irradiated by the same CW laser power, NC membrane had a higher steady-state temperature increase than coverslip. However, increasing laser power can induce thermal damage. The pyrolysis of NC membrane started in the temperature range of 180 ~ 240 $^{\circ}C$.^1^ In contrast, the glass coverslip is less prone to thermal damage whose melting temperature is approximately 1400 ~ 1600 $^{\circ}C$. Thus, the coverslip can tolerate much higher (144-fold) CW laser power without thermal damage than NC membrane, as shown in Figure S5d. This indicates that should the laser power get high enough, the thermal signal from GNP-coverslip would surpass the maximum thermal signal achievable by GNP-NC membrane.


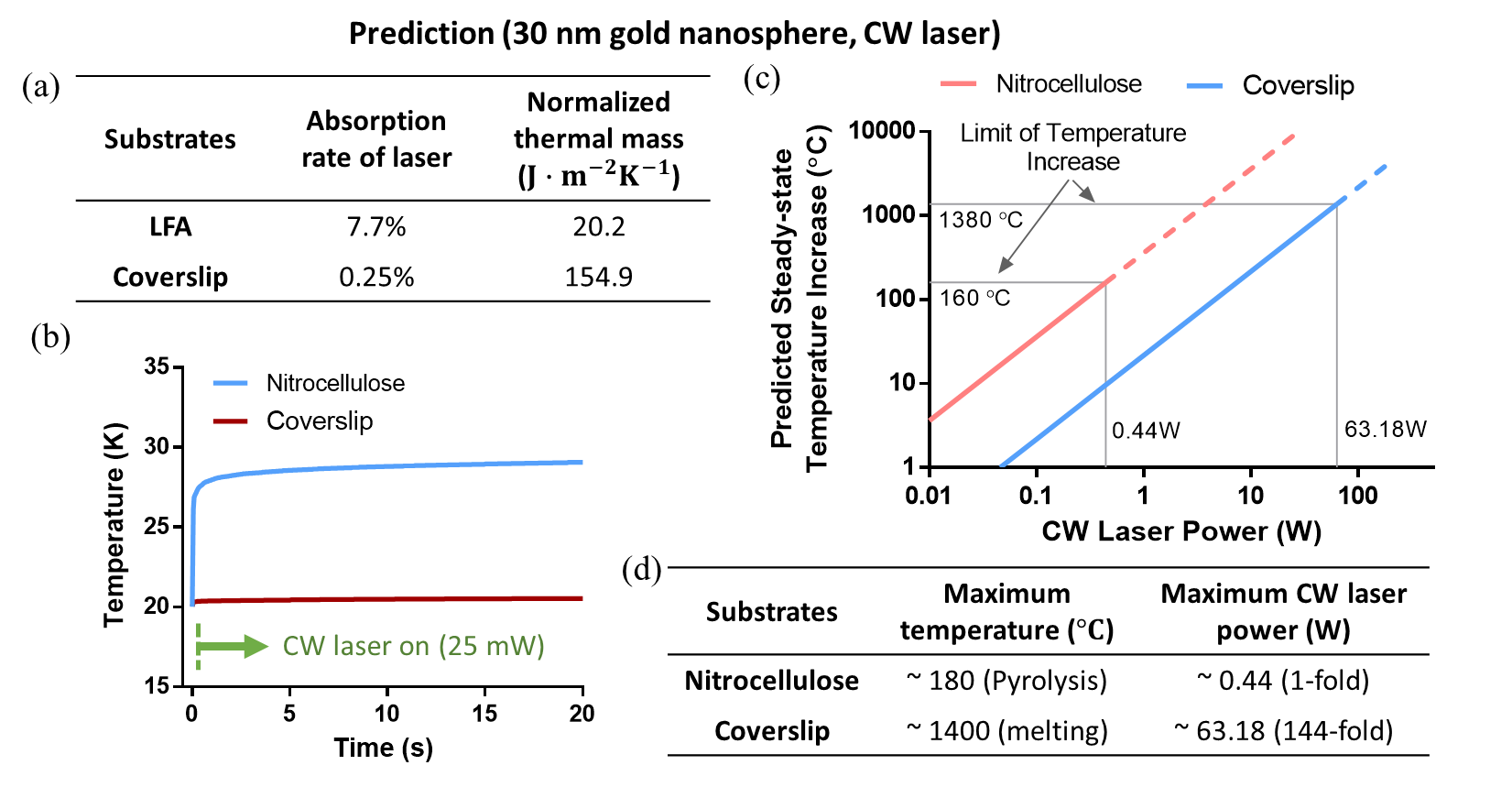


**Figure S5.** **Modeled thermal responses from nitrocellulose (NC) membrane *vs.* coverslip under CW laser heating.** The 30 nm gold nanospheres (GNSps) in NC membrane or on coverslip were set at the same projected surface concentration and heated by the same continuous-wave (CW) TCA. (a) The total absorption rates of the laser energy fluence by GNSp-NC membrane and GNSp-coverslip systems and thermal mass of substrates normalized by the same projected surface area. (b) Temperature responses of different systems at the central heated area (50 $\mu m$ size), mimicking the recordings of an IR camera in CW laser TCA. (c) Predicted steady-state temperature increase based on the model for different systems at increasing CW laser power. (d) Predicted maximum CW laser power for different systems without thermal damage. In the model, it was assumed that the laser power was 25 mW, with a $1/e^{2}$radius of 0.05 mm and that the environmental temperature was 20 $^{\circ}C$. For NC membrane, the GNSs’ projected surface concentration = volumetric concentration$\times$membrane thickness; for coverslip, it equaled to surface concentration.

Additionally, comparison of pulsed laser heating of different substrate systems was also predicted. The same assumed systems as above (*i.e.*, 30 nm GNSp-loaded NC membrane *vs*. coverslip) were used to estimate peak temperature increase under pulsed laser heating. As shown in Figure S6, although NC membrane could have a higher temperature increase than coverslip for the same pulse energy, it was prone to thermal damage. When applying high enough laser energy fluence which cannot be tolerant with NC membrane, the thermal signal of GNP-coverslip would surpass the maximum signal of GNSp-NC membrane. The predicted maximum laser pulse energy tolerance by GNSp-coverslip was 2065-fold higher than GNSp-NC membrane. Note that although the maximum laser power or energy can vary in values for different GNPs and lasers, the trend of comparison between substrate systems is expected to apply to various GNPs and lasers.


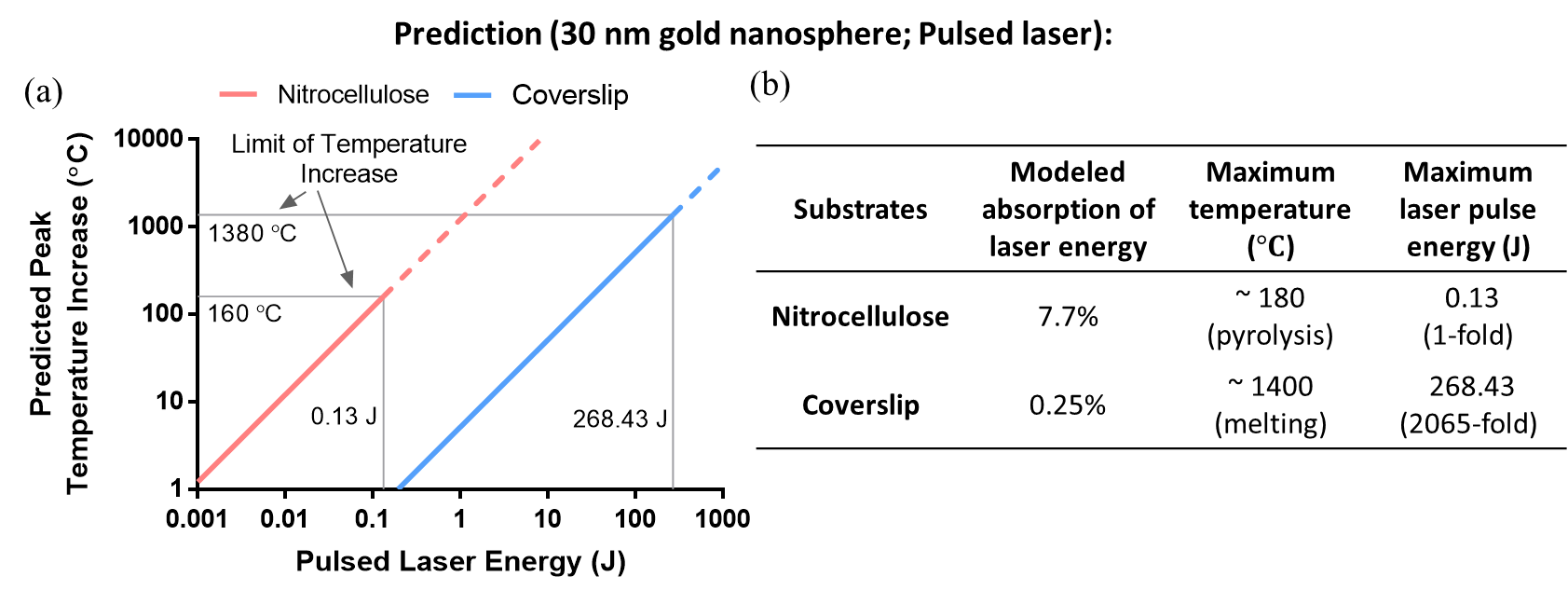


**Figure S6.** **Predicted peak temperature from nitrocellulose (NC) membrane *vs.* coverslip under pulsed laser heating.** The prediction was based on the same assumed systems (*i.e.*, 30 nm gold nanosphere-loaded substrates), laser absorption rates, and boundary conditions as modeled in Figure S5. Both systems were assumed as being read by 20 ms pulsed laser TCA at different pulse energy outputs. (a) Predicted peak temperature increase of substrate systems at different pulsed laser heating. (b) Predicted maximum laser pulse energy for these assumed systems without thermal damages.

## S4.2 Modeling Methods

### (1) Modeling CW Laser Heating of 30 nm GNSps in Nitrocellulose

To study the effects of substrates on thermal signals, optical and thermal modeling was carried out to calculate the temperature responses of GNSp-loaded NC membrane and coverslip as model test regions in immunoassays during TCA reading. For the GNSp-loaded NC membrane, the specific absorption rate (SAR, or the heat source) during laser heating was modeled by the Monte Carlo method.^2^ Since the NC membrane has a multi-layer structure (from top to bottom: opaque NC membrane, transparent polyester backing, and opaque polystyrene backing), a multilayered Monte Carlo package by Lihong Wang et al. was applied (Release 5: MCML 1.2.2 & CONV 1.1).^2–5^ Its validation was provided in subsection S4.2 (2). The input parameters for each layer included the layer thickness (*d*), refractive index (*n*), absorption coefficient ($\mu_{a}$), scattering coefficient ($\mu_{s}$), and anisotropy factor (*g*). The thickness of layers and optical properties of membraned loaded with 0.15 nM 30 nm GNSps were measured in a previous study.^6^ According to Beer’s law, light can hardly go through the membrane with a thickness of 50 $\mu m$ and extinction coefficient of ~3000 $cm^{-1}$ (transmittance <0.0001%). Thus, only one layer was applied in the Monte Carlo calculation. The measured optical properties of the membrane^6^ are summarized in Table S1. The power and spot size of light irradiation onto the membrane for the CW laser TCA were assumed according to the previous measurement when the light went through a planoconvex lens, as shown in Table S2.^7^

The SAR (*i.e.*, heat source) obtained from Monte Carlo modeling was then imported into COMSOL for heat transfer simulation. The thermal properties of the NC membrane are summarized in Table S1. In this simulation, an axial symmetric 2D structure with multiple layers was used to model the irradiated and nearby parts of an NC membrane. The thickness of layers was measured by caliper, as shown in Table S1^6^, and the radius of modeled part ($R_{0}$) of the NC membrane was 2.2 mm. Natural convection was assumed as a boundary condition for the NC membrane, whose heat transfer coefficient was assumed as 10 $W\cdot m^{-2}\cdot K^{-1}$ with ambient temperature at 20 $^{\circ}C.$The initial temperature of the surface at $R_{0}$ was assumed to be equal to the ambient temperature. Confirmative simulation was pre-conducted to ensure the modeling results were irrelevant to $R_{0}$ and grid size.

Based on the above model, steady-state temperature increase ($\Delta T_{ss}$) under different CW laser powers was predicted for both GNSp-loaded coverslip and NC membrane systems. During steady-state heating, the heat generation due to laser irradiation ($\dot{Q}$) equaled to heat dissipation as below.

$$\dot{Q}=U\cdot A\cdot\Delta T_{ss} (S1)$$

where *U* was the average overall heat transfer coefficient; *A* was the overall surface area corresponding to *U*. The value of $U\cdot A$ was estimated by the above COMSOL simulation and was assumed the same for the same GNSp-substrate with increasing laser power.

**Table S1.** Thermal properties and thickness of different layers of a lateral flow immunoassay (LFA).^6^

| Material | Density  ($kg\cdot m^{-3}$) | Thermal conductivity  (W$\cdot m^{-1}\cdot K^{-1}$) | Heat capacity  ($kJ \cdot\mathrm{kg}^{-1}\cdot K^{-1}$) | Thickness  ($\mu m$) |
| --- | --- | --- | --- | --- |
| Top: Membrane^8–10^  (air + nitrocellulose) | 344.61 | 0.078 | 1.174 | 50 |
| Middle: Nitrocellulose backing^11^ (Mylar film) | 1390 | 0.181 | 1.172 | 90 |
| Bottom: LFA backing^12,13^ (Polystyrene) | 1054 | 0.146 | 1.215 | 500 |

**Table S2.** Optical properties of membrane and parameters of light irradiation on the membrane of a nitrocellulose membrane loaded with 0.15 nM 30 nm gold nanospheres.^6^

| Parameters | Values |
| --- | --- |
| Refractive index | 1.45 |
| Absorption coefficient | 0.46 $cm^{-1}$ |
| Scattering coefficient | 2778.3 $cm^{-1}$ |
| Anisotropy factor | 0.6 |
| Irradiation power^7^ | 25 mW |
| $1/e^{2}$ radius of irradiation^7^ | 50 $\mu m$ |

### (2) Validation of Monte Carlo Modeling

To validate the effectiveness of the Monte Carlo modeling package used above, two extreme cases (*i.e.*, no scattering and high scattering) were modeled by this method, with distributions of laser energy fluence compared to the analytical solutions from the well-established reference by AJ Welch et al.^14^ It was assumed that a semi-infinite turbid slab was irradiated by a collimated laser with a matched refractive index at the boundaries.

For the no-scattering case, which assumed that scattering coefficient $\mu_{a}=10 cm^{-1}$ and scattering coefficient $\mu_{s}=0$, the distribution of laser energy fluence followed Beer-Lambert law and normalized by the maximum inlet rate as:^14^

$$\phi\left( z \right)=exp \left( -\left( \mu_{a}+\mu_{s} \right)*z \right) (S2)$$

For a high-scattering case, it was assumed that $\mu_{a}=0.1 cm^{-1}$, $\mu_{s}=100 cm^{-1}$, and anisotropy factor *g*=0.1. Since $\mu_{a}\ll\mu_{s}(1-g)$, the diffusion theory can be applied in this high-scattering case, whose analytical solution for the laser energy fluence was:

$$\phi\left( z \right)=\frac{5}{1+2*\frac{\mu_{a}}{\mu_{eff}}}\exp\left( -\mu_{eff}*z \right)-2*\exp\left( -\mu_{t}*z \right) (S3)$$

where the extinction coefficient $\mu_{t}=\mu_{a}+\mu_{s}$; the effective extinction coefficient $\mu_{eff}=\sqrt{3*\mu_{a}*(\mu_{a}+\left( 1-g \right)*\mu_{s})}$.

For both cases, Figure S7 shows comparable laser energy fluence normalized by their maximum between Monte Carlo modeling results and analytical solutions from AJ Welch et al.^14^, thus validating the modeling package.


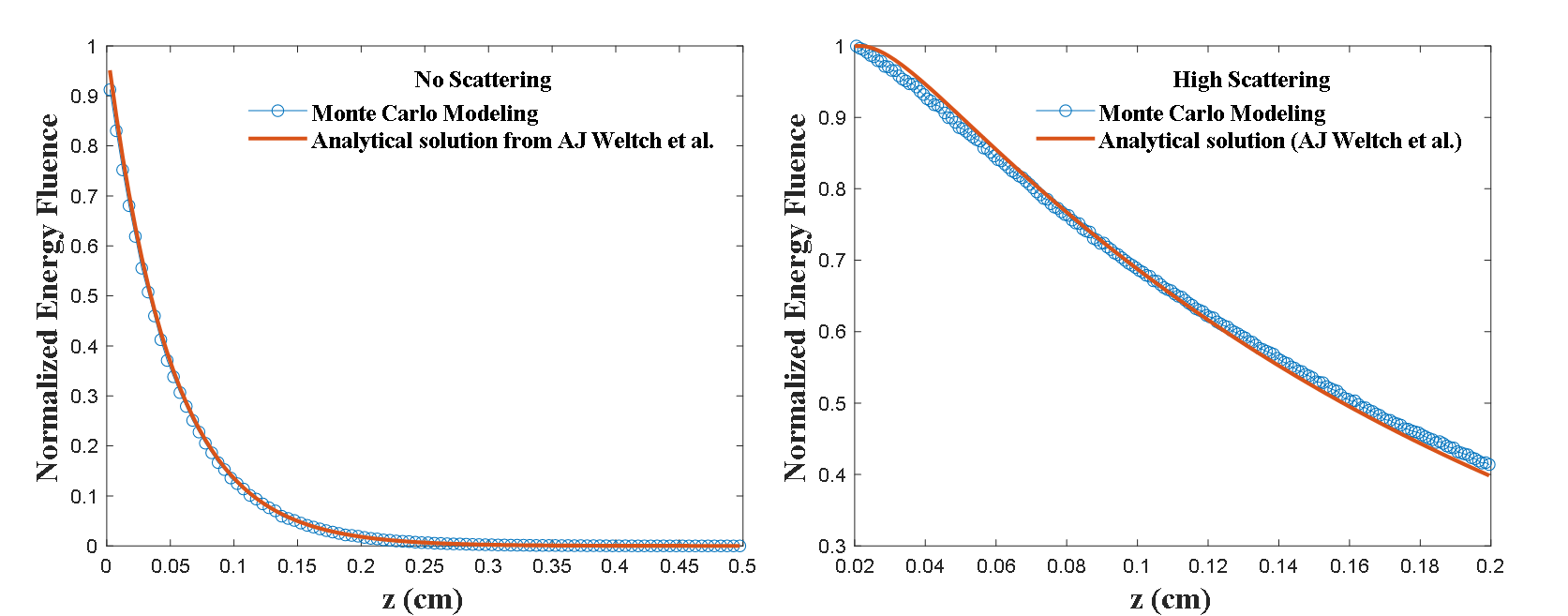


**Figure S7.** Validation of the Monte Carlo modeling by comparison with analytical solutions from AJ Welch et al. ^14^

### (3) Modeling CW Laser Heating of 30 nm GNSps on Coverslip

The coverslip, made of silica, had a thickness of 0.14 mm. The thermal properties of silica glass were included in COMSOL. Since the GNSps were on the coverslip, the SAR was applied as a boundary heat source during heat transfer modeling in COMSOL. The SAR was calculated as

$$SAR=N*C_{abs}*I \left[ W\cdot mm^{-2} \right] (S4)$$

where *N* was the surface concentration of GNSps on coverslip. *N* was set the same as the projected surface concentration of GNSps used in NC membrane modeling ($\sim1.7\times{10}^{6} mm^{-2}$), which was calculated by measuring the diameter of the GNSp dot on the membrane when pipetting a certain volume of GNSp solution. The $C_{abs}$ was the absorption cross section of 30 nm GNSp at 532 nm, which equaled 1450 nm^2^ according to a previous study and Mie theory calculation.^15,16^ The $I$ was light irradiation intensity, which followed Gaussian distribution, as below, with $1/e^{2}$ radius and power the same as those in Table S2:

$$I\left( r \right)=6.37\times{10}^{-6}\exp\left( -\frac{r^{2}}{1.25\times{10}^{-9}} \right) \left[ W\cdot mm^{-2} \right] (S5)$$

where *r* was the radial axis in cylindrical coordinate in heat transfer modeling in COMSOL. Other boundary conditions were the same as those for NC membrane modeling.

### (4) Predicting Pulsed Laser heating of 30 nm GNSps in Nitrocellulose or on Coverslip

The peak temperatures for different substrates were also predicted under different pulse energy outputs. During the pulsed laser heating, thermal relaxation time scales ($\tau$) within the laser spot area for different substrates were estimated by

$$\tau=\frac{d^{2}}{27\alpha} (S6)$$

where d was the characteristic length which was estimated as the laser spot size (2 mm); $\alpha$ was thermal diffusivity of substrates. Table S3 estimated $\tau$ for both substrates, both of which were longer than the laser pulse widths ($\leq$20 ms). This indicated thermal confinement during pulsed laser heating. Therefore, lumped system analysis as below was used to estimate the mean peak temperature increase ($\Delta T_{peak}$) of laser-irradiated spots for both substrates under increasing laser pulse energy ($Q$).

$$Q=m\cdot C_{p}\cdot\Delta T_{peak} (S7)$$

where *m* and $C_{p}$ were mass and heat capacity for the systems of interest, respectively.

The absorption rate of irradiated pulsed laser energy was assumed the same as modeled results from CW laser heating cases. The environmental temperature was assumed as 20 $^{\circ}C$.

**Table S3.** Estimated thermal relaxation times for nitrocellulose (NC) membrane and coverslip during pulsed laser heating.

| Substrates | Thermal diffusivity  ($m^{2}\cdot s^{-1}$) | Laser spot size (mm) | Thermal relaxation time (ms) |
| --- | --- | --- | --- |
| Nitrocellulose | 1.9E-7 | 2 | 768 |
| Coverslip (glass) | 3.4E-7 | 2 | 436 |

# S5. Scale Analysis for Microfluidic Assay (MIA)

To evaluate MIA’s assay performance, its capturing of analytes with GNP labels at the test region was estimated and compared with conventional lateral flow immunoassays (LFAs). The accumulated concentration of captured labels relied on the reactants’ concentrations, assay time, reaction rate, and surface area. For comparison, both MIA and LFA were assumed to have the same immunoreactions with the same reactants’ concentrations and assay time.

The reaction rates were compared by scale analysis of reaction and flow kinetics in LFA and MIA. Specifically, the Péclet number (*Pe*$=\frac{convection rate}{diffusion rate}$) and Damköhler numbers (*Da*$=\frac{reaction rate}{diffusion rate}$) were estimated (Table S4), with the label assumed as 100 nm gold nanospheres^15^ and the capture antibody assumed to be precoated at the same high surface concentration (*i.e.*, the local surface density of antibodies without consideration of surface area nor 3D structure, ${10}^{-8} \mathrm{mol}m^{-2}$).^17^ Table S4 presents the characteristic lengths and flow velocities of fluid in MIA and LFA based on previous studies.^15,18^ It shows that MIA had a slower diffusion rate than the convection rate ($Pe\gg1$), while LFA had comparable convection and diffusion rate ($Pe\sim1$). Regarding reaction, the LFA had comparable surface reaction and mass diffusion rates of reactants ($Da\sim1$), indicating that the reaction rate in LFA could be near its optimal achievable rate with adequate reactants fed by diffusion and convection. In contrast, the MIA was diffusion-limited ($Da\gg1$) (i.e., diffusion rate $\ll$ reaction rate); the reaction rate could be lower than its optimal achievable rate due to inadequate reactants from diffusion. The reaction rate in MIA could thus be lower than that in LFAs. The capture rate of target analytes and GNP labels at the test region of MIA could also be lower than that in LFAs.

Additionally, the accumulation of captured labels also depended on the available surface area for reaction. The LFA had porous nitrocellulose (NC) membrane to accommodate reactions, with density and surface area estimated as 1.23 $g cm^{-3}$ and 13.381 $m^{2}g^{-1}$.^19,20^ With an assumed thickness of 0.05 mm, the ratio of overall surface area to the projected area was about 823 for the NC membrane. This suggested that for the same area of testing regions, the LFA could have an 823-fold larger surface area for reactions than MIA when assuming that only the bottom surface of the microfluidic channel was used for surface reaction and signal amplification. LFAs could therefore have an even higher capture rate of labels at test regions than MIAs, leading to a higher sensitivity in diagnosing analytes. To compensate for it, advanced surface modification on MIA, such as 3D polymer coating, might be needed to enable higher loading efficiency of capture antibodies and thus better immunoreaction efficacy. Also, signal amplification, such as TCA, could be used to enhance label detection capability and improve sensitivity. Still, MIAs have the advantage of tolerance for larger targets (e.g., exosomes from cells) and less need for sample volume than LFAs. Although both improvements will be needed for reader and assay design, MIA with ultrafast TCA will still be promising for future ultrafast and sensitive point-of-care diagnostics when there is need for testing large biomarkers, multiplexing, and high throughput.

**Table S4.** Scale analysis for reaction and flow kinetics in a microfluidic immunoassay (MIA) and lateral flow immunoassay (LFA).

| **Parameters** | **Units** | **MIA** | **LFA** |
| --- | --- | --- | --- |
| Effective Diffusivity ($D_{e}$) | $m^{2}s^{-1}$ | $3.5\times{10}^{-12}$ | $3.5\times{10}^{-12}$ |
| Characteristic convection length (*L*) | m | 0.01 | $5\times{10}^{-4}$ |
| Characteristic diffusion length (*R*) | m | $6.7\times{10}^{-5}$ | $5\times{10}^{-6}$ |
| Velocity (*U*) | m/s | 0.01 | $1.8\times{10}^{-4}$ |
| Effective forward reaction constant ($k_{on}^{'}$) | $m^{3}\mathrm{mo}l^{-1} s^{-1}$ | 137 | 137 |
| Concentration of capture antibody ($C_{R}$) | $\mathrm{mol}m^{-2}$ | ${10}^{-8}$ | ${10}^{-8}$ |
| $Pe=UR^{2}$/${LD}_{e}$ |  | $1.3\times{10}^{3}$ | 1.29 |
| $Da=C_{R}k_{on}^{'}R$/$D_{e}$ |  | 26 | 2 |

*The effective diffusivity described the diffusivity of detection antibody-conjugated gold nanosphere with 100 nm diameter.^15^ The values for *U* for LFA and $k_{on}^{'}$ was referenced from a previous LFA study.^15^ The flow and structure parameters for MIA were estimated based on a previous MIA study.^18^ The diffusion length of MIA was featured by the hydrodynamic diameter of its channel, while the convection length was the total channel length for each detection region, assumed to be 10 mm. Similarly, the diffusion length for LFA was the pore size of the membrane, while the convection length was the width of the test line in the lateral flow direction, assumed to be 1 mm.

# S6. Additional Figures


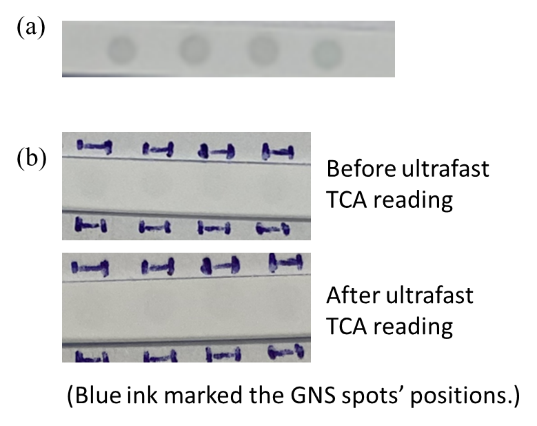


**Figure S8.** (a) Example GNS spots in the nitrocellulose membrane after air drying. (b) Example GNS spots before and after ultrafast TCA reading (170 V, 3ms). The blue ink in panel (b) marked the axial positions of GNS spots in nitrocellulose membrane, which showed very weak color.


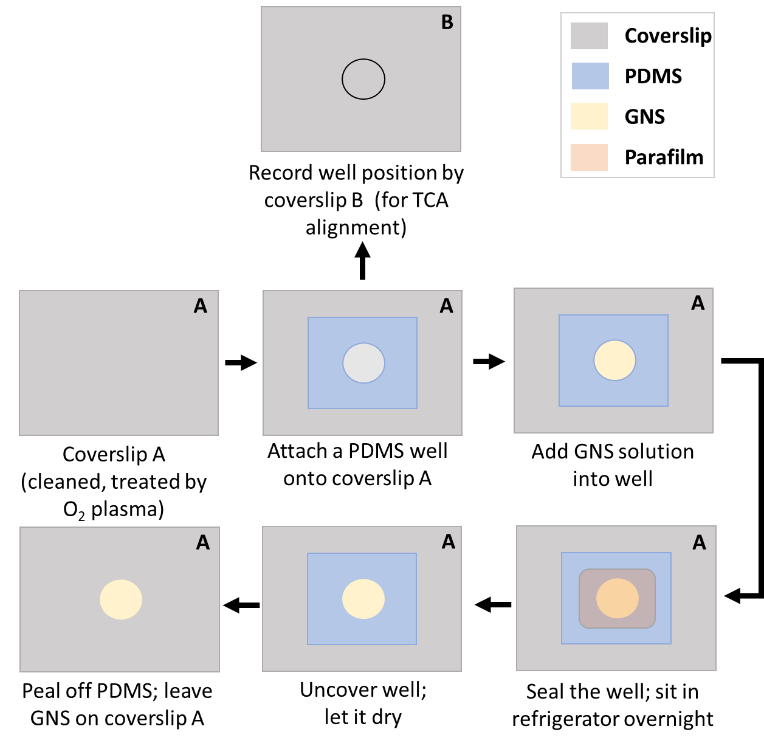


**Figure S9.** The schematic procedures to prepare silica-cored gold nanoshell (GNS) dots on coverslips.


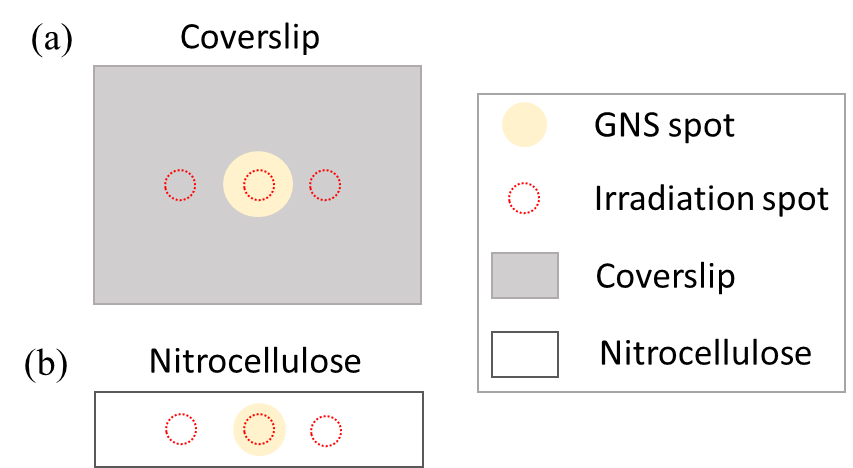


**Figure S10.** The correlation between GNS spot (yellow dots) and irradiation spots (dashed red dots) in ultrafast TCA reading to obtain the thermal signal of a GNS spot: (a) coverslip; (b) nitrocellulose membrane.

# References:

1. Chai, H., Duan, Q., Cao, H., Li, M. & Sun, J. Effects of nitrogen content on pyrolysis behavior of nitrocellulose. *Fuel* **264**, 116853 (2020).

2. Wang, L. & Steven L Jacques, P. D. *Monte Carlo Modeling of Light Transport in Multi-layered Tissues in Standard C*.

3. Wana, L. & Jacaues, S. L. Optimized radial and angular positions in Monte Carlo modeling. *Medical Physics* **21**, 1081–1083 (1994).

4. Wang, L., Jacques, S. L. & Zheng, L. MCML-Monte Carlo modeling of light transport in multi-layered tissues. *Comput. Methods Programs Biomed.* **47**, 131–146 (1995).

5. Wang, L., Jacques, S. L. & Zheng, L. CONV - Convolution for responses to a finite diameter photon beam incident on multi-layered tissues. *Comput. Methods Programs Biomed.* **54**, 141–150 (1997).

6. Wang, Y. Measurement and Application of Heat Generation from Gold Nanoparticle Systems under Laser Irradiation in Biomedicine. (University of Minnesota, 2019).

7. Qin, Z. Laser Induced Gold Nanoparticle Heating: Thermal Contrast in Lateral Flow Immunoassays. *ProQuest Diss. Theses* 237 (2014).

8. Charles, A. *Harper, Handbook of plastics, elastomers, and composites*. (New York: McGraw-Hill, 1992).

9. Mark, J. E. *Physical Properties of Polymers Handbook*. (New York: Springer, 2007).

10. G. M. Brauer & Horowitz, E. *Analytical Chemistry of Polymers, part III*. (John Wiley & Sons, 1962).

11. Idicula, M. *et al.* Thermophysical properties of natural fibre reinforced polyester composites. *Compos. Sci. Technol.* **66**, 2719–2725 (2006).

12. Gaur, U. & Wunderlich, B. Heat capacity and other thermodynamic properties of linear macromolecules. II. Polyethylene. *J. Phys. Chem. Ref. Data* **10**, 119–152 (1981).

13. Yu, S., Hing, P. & Hu, X. Thermal conductivity of polystyrene–aluminum nitride composite. *Compos. Part A Appl. Sci. Manuf.* **33**, 289–292 (2002).

14. Welch, A. J. & Van Gemert, M. J. *Optical-thermal response of laser-irradiated tissue*. (Springer, 2011).

15. Zhan, L. *et al.* The Role of Nanoparticle Design in Determining Analytical Performance of Lateral Flow Immunoassays. *Nano Lett.* **17**, 7207–7212 (2017).

16. Oldenburg, S. J. Light scattering from gold nanoshells. *Rice Univ.,Houston,TX,USA. FIELD URL:* (Rice University, 2000).

17. Liu, Y. *et al.* fM–aM Detection of the SARS-CoV-2 Antigen by Advanced Lateral Flow Immunoassay Based on Gold Nanospheres. *ACS Appl. Nano Mater.* **4**, 13826–13837 (2021).

18. Chin, C. D. *et al.* Microfluidics-based diagnostics of infectious diseases in the developing world. *Nat. Med.* **17**, 1015–1019 (2011).

19. Ahmad, A. L., Low, S. C. & Shukor, S. R. A. Effects of membrane cast thickness on controlling the macrovoid structure in lateral flow nitrocellulose membrane and determination of its characteristics. *Scr. Mater.* **57**, 743–746 (2007).

20. Tang, R. H., Liu, L. N., Zhang, S. F., Li, A. & Li, Z. Modification of a nitrocellulose membrane with cellulose nanofibers for enhanced sensitivity of lateral flow assays: application to the determination of Staphylococcus aureus. *Microchim. Acta* **186**, 831 (2019).
